# Search for serum biomarkers in patients with bipolar disorder and major depressive disorder using metabolome analysis

**DOI:** 10.3389/fpsyt.2023.1251955

**Published:** 2023-09-06

**Authors:** Xiao-Li Sun, Li-Na Ma, Zhen-Zhu Chen, Yan-Bing Xiong, Jiao Jia, Yu Wang, Yan Ren

**Affiliations:** ^1^Department of Psychiatry, Shanxi Bethune Hospital, Shanxi Academy of Medical Sciences, Tongji Shanxi Hospital, Third Hospital of Shanxi Medical University, Taiyuan, China; ^2^Tongji Hospital, Tongji Medical College, Huazhong University of Science and Technology, Wuhan, China; ^3^Changzhi Mental Health Center, Changzhi, China

**Keywords:** bipolar disorder, major depressive disorder, metabonomics, biomarker, nuclear magnetic resonance

## Abstract

**Objective:**

Bipolar disorder (BD) and major depressive disorder (MDD) are two common psychiatric disorders. Due to the overlapping clinical symptoms and the lack of objective diagnostic biomarkers, bipolar disorder (BD) is easily misdiagnosed as major depressive disorder (MDD), which in turn affects treatment decisions and prognosis. This study aimed to investigate biomarkers that could be used to differentiate BD from MDD.

**Methods:**

Nuclear magnetic resonance (NMR) spectroscopy was performed to assess serum metabolic profiles in depressed patients with BD (*n* = 59), patients with MDD (*n* = 14), and healthy controls (*n* = 10). Data was analyzed using partial least squares discriminant analysis, orthogonal partial least squares discriminant analysis and *t*-tests. Different metabolites (VIP > 1 and *p* < 0.05) were identified and further analyzed using Metabo Analyst 5.0 to identify relevant metabolic pathways.

**Results:**

The metabolic phenotypes of the BD and MDD groups were significantly different from those of the healthy controls, and there were different metabolite differences between them. In the BD group, the levels of 3-hydroxybutyric acid, n-acetyl glycoprotein, β-glucose, pantothenic acid, mannose, glycerol, and lipids were significantly higher than those in the healthy control group, and the levels of lactate and acetoacetate were significantly lower than those in the healthy control group. In the MDD group, the levels of 3-hydroxybutyric acid, n-acetyl glycoprotein, pyruvate, choline, acetoacetic acid, and lipids were significantly higher than those of healthy controls, and the levels of acetic acid and glycerol were significantly lower than those of healthy controls.

**Conclusion:**

Glycerolipid metabolism is significantly involved in BD and MDD. Pyruvate metabolism is significantly involved in MDD. Pyruvate, choline, and acetate may be potential biomarkers for MDD to distinguish from BD, and pantothenic acid may be a potential biomarker for BD to distinguish from MDD.

## 1. Introduction

Major depressive disorder (MDD) and bipolar disorder (BD) are two different psychiatric disorders with commonly overlapping symptoms. According to the Diagnostic and Statistical Manual of Mental Disorders, Fifth Edition (DSM-5), MDD is characterized by the presence of major depressive episodes, whereas patients with BD have depressive episodes preceding or following a mild manic or manic episode [American Psychiatric Association (1)]. Typically, patients with BD cycle between manic, depressive, and normal mood episodes; however, the depressive phase of BD occurs more frequently than the hypomanic or manic phase (2); BD I is characterized by one or more manic episodes or mixed episodes and major depressive episodes; BD II is characterized by recurrent depressive episodes and hypomanic episodes, but no manic episodes; but hypomania is in fact difficult to detect in clinical practice, therefore leading to delayed diagnosis or misdiagnosis of BD as MDD, particularly bipolar II disorder (BD II) (3, 4).

Delayed diagnosis and misdiagnosis of bipolar disorder may result in the use of antidepressant monotherapy, creating an increased risk of patients moving to a hypomanic or manic episode (5, 6). Approximately 40–60% of patients with bipolar disorder are initially diagnosed with MDD, and an accurate diagnosis or treatment of bipolar disorder may be delayed by 5–10 years (7). Definitive diagnosis and treatment are critical to improving psychiatric disorders’ symptoms and functional prognosis. On the contrary, under-treatment and delayed treatment increase both the direct and indirect economic costs associated with BD, result in increase individual suffering, and compromise overall prognosis (8). In fact, a few pre-existing situations in MDD and BD diagnosis may add extra challenges to their under-treatment. Particularly, to date, the pathophysiological mechanisms of MDD and BD remain unclear, the objective biomarkers for differentiating these two disorders are still lacking, and many clinicians have been using subjective identification to diagnose MDD and BD based on symptom clustering from standardized structured diagnostic interviews. Therefore, searching for specific biomarkers to differentiate BD from MDD is crucial.

Metabolomics is a new addition to the field of histology, focusing on measuring the downstream effects of environmental, genomic, and proteomic variation in individuals by identifying and quantifying small molecules called metabolites (9). By assessing the abundance and type of metabolites detected, metabolomics can provide a functional readout of the cellular state within an individual and help us identify biochemical signatures or biomarkers specific to different diseases (10). Currently, metabolomics has unique and proven advantages in the development of biomarkers for several diseases (11, 12). The serum is used in metabolomics studies and has been used for many common diseases such as cardiovascular injury, diabetes, Parkinson’s disease, and depression (13). The analytical techniques commonly used in metabolomics studies are nuclear magnetic resonance (NMR) and mass spectrometry (MS) (14). NMR has been widely used in current metabolomics research because of its advantages such as fast test speed, non-invasive and comprehensive metabolite information coverage.

Using an NMR metabolomics approach, our group has conducted several studies on the search for BD biomarkers in the early stages (15–17). These identified biomarkers were able to accurately distinguish BD patients from healthy controls. However, the effectiveness of these biomarkers in discriminating BD from MDD remains unclear. To avoid misdiagnosis, previous studies have identified a number of candidate biomarkers to differentiate between MDD and BD patients (18–21). However, these putative biomarkers have not been used in clinical practice due to the high heterogeneity and overlapping dimensions between MDD and BD.

Therefore, in the present study, we used an NMR metabolomics approach to analyze serum metabolic phenotypes in BD, MDD, and healthy controls to initially explore biomarkers that may help differentiate BD and MDD and to further understand the pathophysiology of both diseases.

## 2. Materials and methods

### 2.1. Subject recruitment

Ethics approval for this study is held by the medical ethics committee of Shanxi Bethune Hospital (the Approval Notice Number: YXLL-2020-001). All subjects enrolled in the study gave their written informed consent. A pair of licensed, experienced psychiatrists were in charge of the recruiting procedure.

The current study was conducted at the Department of Psychiatry in Shanxi Bethune Hospital from July 2019 to February 2021. Fifty-nine patients with BD who fulfilled the bipolar depression criteria of the DSM-5 (Diagnostic and Statistical Manual of Mental Disorders, Fifth Edition) were recruited. MDD subjects were recruited from the same site and during the same time period. Fourteen candidates of MDD, who were diagnosed with MDD (Hamilton Depression Scale rating ≥ 17) using the Structured Clinical Interview, were recruited. Patients with any physical or other mental disorders were excluded, as were patients who had substance abuse issues. To reduce the risk of misdiagnosed BD subjects among the MDD subjects included in this study, a licensed psychiatrist in our department who has specialized in the diagnosis and treatment of BD and MDD for several years systematically applied the validated structured interviews to diagnose each patient.

During the same time period, healthy controls (HC) were recruited from the Medical Examination Center in the same hospital. The candidates, who had no history of neurological, systemic medical illness, or DSM-IV Axis I/II illness, were recruited. Finally, 10 HC subjects were included. The demographic and clinical characteristics of the included subjects are shown in Table 1. The age of onset was defined as the age (in years) at which a patient first experienced the emotional symptoms described to a psychiatrist from the study’s research team. The duration of illness was the period (in months) from the first onset to the time of enrollment.

**TABLE 1:** Clinicodemographic characteristics of the participants.

Variable	Group			*p*-value
	**BD (*N* = 59)**	**MDD (*N* = 14)**	**HC (*N* = 10)**	
Age (years)	27.73 ± 10.84	33.12 ± 15.20	28.5 ± 3.10	0.271
BMI	22.40 ± 3.63	22.79 ± 3.67	21.6 ± 3.62	0.271
Sex (M/F)	22/37	7/7	3/7	0.570
Onset age (years)	22.10 ± 9.60	29.00 ± 15.56	–	0.132
Duration of illness (months)	39.39 ± 42.52	33.93 ± 36.38	–	0.659
The total scores of HAMD-24	29.05 ± 7.60	21.93 ± 10.37	–	0.058

Data are expressed as the mean (standard deviation).

BD, bipolar disorder; MDD, major depressive disorder; HC, healthy control; M/F, male/female; BMI, body mass index.

### 2.2. Sample collection

The blood samples of all subjects were collected by medical professionals at the Shanxi Bethune Hospital in the morning after a 12-h fast. The blood was mixed inverted after clotting and stored for 30 min at room temperature (about 20°C) before centrifugation at 3,000 rpm for 15 min. Then, the serum was collected and stored in a −80°C refrigerator for future use.

### 2.3. NMR acquisition

The serum samples were thawed in ice water, and 450 μL supernatant was removed and placed into an EP tube, 350 μL D2O was added, and the mixture was centrifuged at 4°C for 20 min (13000 r/min). Then, 600 μL supernatant was transferred to a 5 mm NMR tube and stored at 4°C until the NMR test. NMR was performed using a Bruker 600 MHz AVANCE III NMR spectrometer, with Carr-Purcell Meiboom-Gill pulse sequence, and the following parameter settings: free induction attenuation (64K data points), self-axonal relaxation delay (320 ms), 64 scans.

### 2.4. Data processing

All the acquired ^1^H NMR spectra were manually phased, and the baseline was set using MestReNova software (Mestrelab Research, Santiago de Compostella, Spain). All the serum ^1^H NMR profiles were Fourier transformed and phase baseline adjusted. Chemical shift correction was performed on the profiles based on creatinine (δ 3.04, -CH3). The region of δ 4.70–5.20 ppm was excluded due to residual water. The data were then normalized to the total sum of the spectra.

### 2.5. Statistical analysis

Partial least square discriminant (PLS-DA) analysis was performed, followed by orthogonal partial least square discriminant (OPLS-DA) analysis using SIMCA-P 14.1. To determine the pathways involved in differentially occurring metabolites, they were further introduced to MetaboAnalyst 5.0^1^ to perform pathway analysis using the human pathway library. Pathways were screened according to the *p*-values of pathway enrichment and impact values of pathway topology analysis.

The *t*-test was used to detect and identify differences in markers between BD and MDD. All clinical scale data were expressed as the mean ± SD. Continuous variables were analyzed using a one-way analysis of variance, while categorical variables were analyzed using the Chi-square test. All statistical analyses were performed using SPSS 20.0 (IBM, Chicago, IL, United States). A *p*-value of ≤0.05 was considered statistically significant for demographic analysis.

## 3. Results

### 3.1. Clinical characteristics

There were no significant differences in age, education, or sex ratio (male/female) among the BD, MDD, and HC. There were also no significant differences between the BD and MDD groups concerning the age at onset, duration of illness, and HAMD scores. Table 1 shows the clinicodemographic data of the participants.

### 3.2. ^1^H NMR spectroscopy data analysis

**FIGURE 1:**
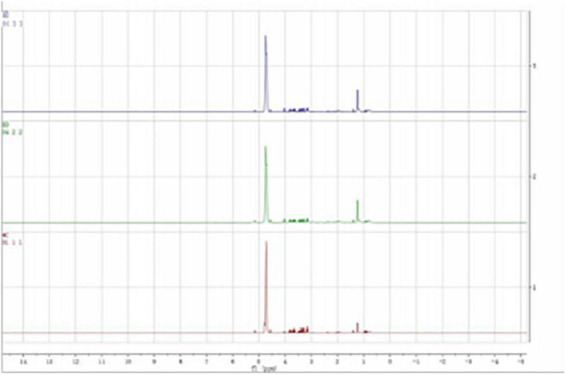
Typical ^1^H NMR spectrum of serum in MDD, BD, and healthy controls groups.

According to previous literature and the NMR data website (HMDB),^2^ the chemical shift, peak shape, and coupling constant of each metabolite were confirmed, and the ^1^H-NMR metabolite maps of the blank, BD, and MDD groups were obtained (Figure 1), from which 29 small-molecule compounds were identified (Table 2).

**TABLE 2:** Peak attribution in ^1^H-NMR spectra of differential metabolites among the three groups.

No	Metabolites	Chemical shift
1	Lipids	0.874 (m)
2	Pantothenate	0.907 (s)
3	Isoleucine	0.949 (t)
4	Leucine	0.961 (t)
5	3-Hydroxybutyric acid	1.21 (d)
6	Lactate	1.33 (d)
7	Acetic acid/Acetate	1.927 (s)
8	O-Acetyl glycoproteins	2.14 (s)
9	Acetoacetate	2.28 (s), 3.44 (s)
10	β-glucose	3.25 (dd, 9.4 Hz, 8.1 Hz)
11	Guanidinoacetate	3.80 (s)
12	Pyruvate	2.37 (s)
13	Histidine	7.04 (s), 7.84 (s)
14	Dimethylglycine	2.92 (s), 3.70 (s)
15	Creatine	3.04 (s), 3.93 (s)
16	Acetylcholine	3.23 (s)
17	Taurine	3.27 (t, *J* = 6.6 Hz), 3.42 (t, *J* = 6.6 Hz)
18	Mannose	5.19 (d, 1.6 Hz)
19	3-D-hydroxybutyrate	1.20 (d)
20	Betaine	3.27 (m)
21	Glycerol	3.67 (m), 3.78 (m)
22	Citrulline	3.73 (s)
23	N-Acetyl glycoproteins	2.05 (s)
24	Glutamate	2.06 (m), 2.14 (m), 2.36 (m)
25	Glutamine	2.14 (m)
26	Acetone	2.23 (s)
27	Acetoacetate	2.28 (s), 3.44 (s)
28	Citric acid/citrate	2.53 (d, 16.1 Hz), 2.70 (d, 16.1 Hz)
29	Choline	3.20 (s), 4.06 (m)

### 3.3. Discriminative model construction

**FIGURE 2:**
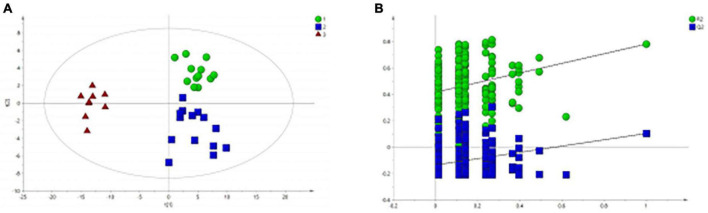
PLS-DA analysis of ^1^H NMR spectra of serum samples from BD, MDD, and HC groups. **(A)** PLS-DA score plots of ^1^H NMR spectra from BD (circle), MDD (square), and HC group (triangle); **(B)** PLS-DA model validation map.

All serum samples were analyzed by ^1^H NMR metabolic profiling using supervised PLS-DA, and the results were shown in Figure 2A, indicating that the HC group was completely separated from the BD and MDD groups. Model verification results are shown in Figure 2B. The results show that the PLS-DA model is effective.

Figure 2A shows that there is no significant difference between the BD group and the MDD group. Therefore, we analyzed and compared the BD group and the MDD group with the HC group respectively to further identify the metabolites changes and analyze their changing trends.

**FIGURE 3:**
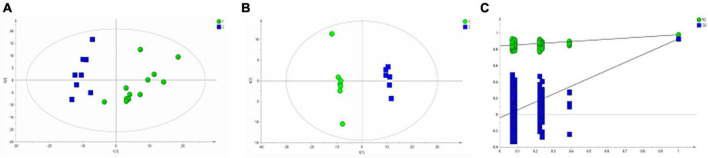
Metabolomics analysis of serum samples from HCs and patients with BD. **(A)** PCA (Principal Component Analysis) model; **(B)** PLS-DA model; **(C)** 200-iteration permutation test map of the PLS-DA model.

**FIGURE 4:**
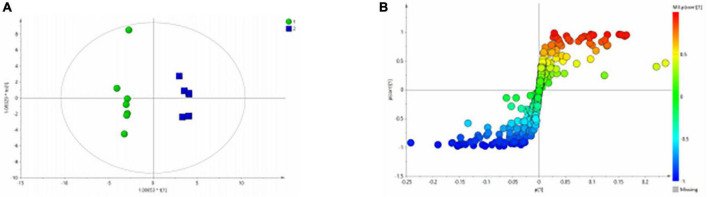
OPLS-DA analysis of ^1^H NMR spectra of serum samples from HCs and patients with BD. **(A)** Score plots of OPLS-DA model; **(B)** S-plot.

Figure 3A shows the differential metabolites of the healthy and BD groups analyzed using PCA (Principal Component Analysis). As shown in the figure, the HC and BD groups were significantly separated, indicating that the model was successfully replicated. To reduce intragroup error, PLS-DA profile analysis was performed for the HC group and BD group (Figure 3B). The groups were significantly separated along T [1], and the model was verified 200 times to prove its validity (Figure 3C). OPLS-DA analysis was performed to reduce the random errors unrelated to the target in the group and to identify the differential metabolites between the blank group and the BD group, as shown in Figure 4A. The differential metabolites with VIP > 1 were identified according to the S-plots (Figure 4B), and an independent sample *t*-test was performed to screen out the metabolites with significant differences (*p* < 0.05, *p* < 0.01).

**FIGURE 5:**
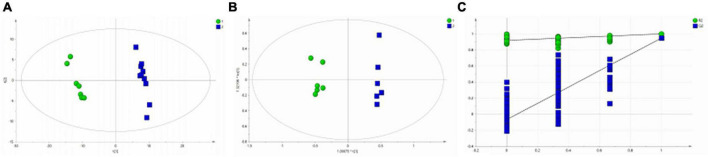
Metabolomics analysis of serum samples from HCs and patients with MDD. **(A)** PCA (Principal Component Analysis) model; **(B)** PLS-DA model; **(C)** 200-iteration permutation test map of the PLS-DA model.

**FIGURE 6:**
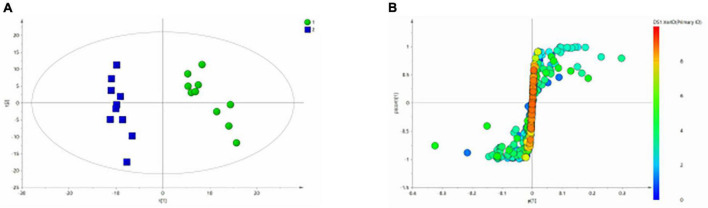
OPLS-Da analysis of ^1^H NMR spectra of serum samples from HCs and patients with MDD. **(A)** Score plots of OPLS-DA model; **(B)** S-plot.

Similarly, the group of MDD was significantly separated from HC group in the PCA (Figure 5A) and PLS-DA model (Figure 5B), which were verified to be valid by the permutation testing (Figure 5C). The score plot of OPLS-DA model (Figure 6A) and the corresponding S-plot (Figure 6B) indicated the differential metabolites (VIP > 1, *p* < 0.05).

### 3.4. Differences in the serum metabolite and metabolic pathways between the BD and HC groups

**FIGURE 7:**
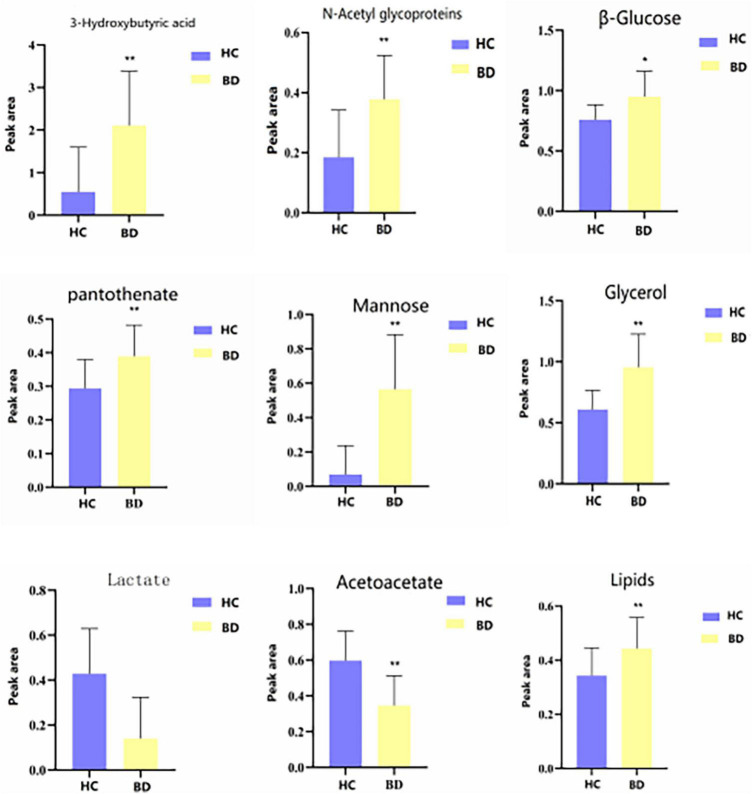
Differential metabolites level in serum ^1^H NMR spectra of the HC and BD groups. Compared with control group, *P < 0.05 and **P < 0.01.

**FIGURE 8:**
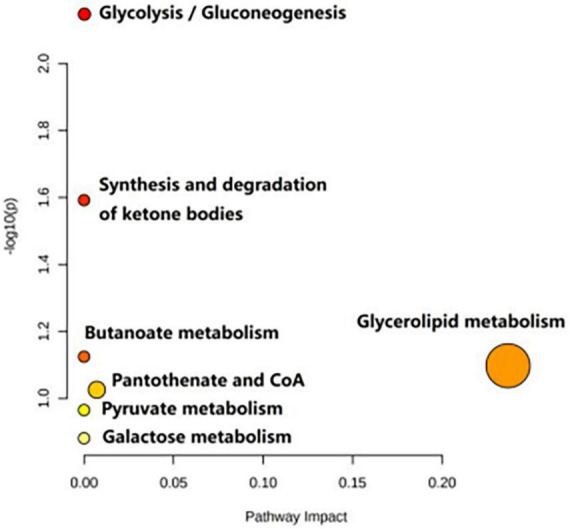
Metabolic pathways analysis of the differential metabolites between the HC and BD groups.

Table 3 shows the influence of potential biomarkers in the BD group. With the HC group as reference and VIP > 1 and *p* < 0.05, nine metabolites differentially expressed in the BD group were identified. 3-hydroxybutyric acid, N-acetyl glycoproteins, β-glucose, pantothenate, mannose, glycerol, and lipids levels were significantly higher in the BD group (*p* < 0.05, *p* < 0.01), while lactate and acetoacetate levels were significantly lower (*p* < 0.05, *p* < 0.01). The relative concentrations of the nine identified metabolites are shown in Figure 7. The results of Metabo Analyst 5.0 analysis are presented graphically as a bubble plot in Figure 8. The darker color and larger size represent higher *p*-values from enrichment analysis and greater impact from pathway topology analysis, respectively. This model is mainly related to: (1) glycolysis/gluconeogenesis; (2) glycerolipid metabolism; (3) synthesis and degradation of ketone bodies; (4) butanoate metabolism; (5) pantothenate and COA; (6) pyruvate metabolism; and (7) galactose metabolism.

**TABLE 3:** Peak area of metabolites in serum ^1^H-NMR spectra of the HC and BD groups.

Metabolites	Peak area after normalization	
	**HC**	**BD**
3- -hydroxybutyric acid	0.549 ± 1.048	2.019 ± 1.272
N- -acetyl glycoproteins	0.185 ± 0.156	0.378 ± 0.145
β-glucose	0.75 ± 0.124	0.95 ± 0.211
Pantothenate	0.293 ± 0.086	0.389 ± 0.092
Mannose	0.069 ± 0.167	0.565 ± 0.317
Glycerol	0.609 ± 0.156	0.954 ± 0.273
Lactate	0.428 ± 0.200	0.139 ± 0.183
Acetoacetate	0.597 ± 0.164	0.346 ± 0.166
Lipids	0.343 ± 0.102	0.443 ± 0.114

### 3.5. Differences in the serum metabolite and metabolic pathway between the MDD and HC groups

**FIGURE 9:**
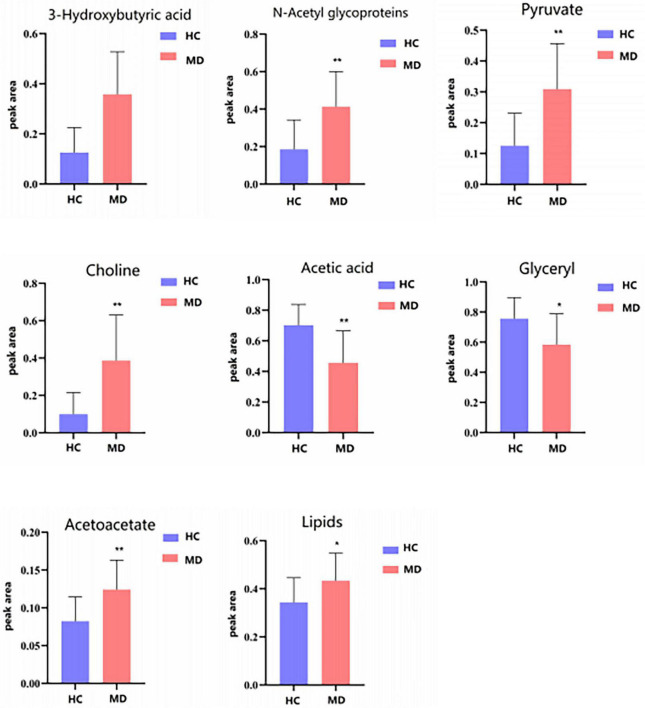
Differential metabolites level in serum ^1^H NMR spectra of the HC and MDD groups. Compared with control group, *P < 0.05 and **P < 0.01.

**FIGURE 10:**
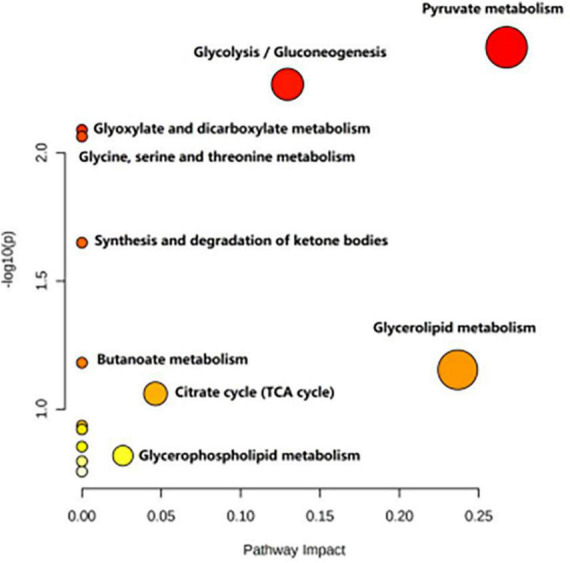
Metabolic pathways analysis of the differential metabolites between the HC and MDD groups.

Table 4 shows the potential biomarkers in the serum of patients with MDD. With the HC group as a reference and VIP > 1 and *p* < 0.05, eight metabolites differentially expressed in the MDD group were identified. 3-Hydroxybutyric acid, N-acetyl glycoproteins, pyruvate, choline, acetoacetate, and lipids levels were significantly higher in the MDD group (*p* < 0.05, *p* < 0.01), while acetic acid and glyceryl level were significantly lower (*p* < 0.05, *p* < 0.01). The relative concentrations of the eight identified metabolites are shown in Figure 9. The results of the MetaboAnalyst 5.0 analysis are shown in Figure 10. This model is mainly related to: (1) pyruvate metabolism; (2) glycolysis/gluconeogenesis; (3) glyoxylate and dicarboxylate metabolism; (4) glycine, serine and threonine metabolism; (5) synthesis and degradation of ketone bodies; (6) glycerolipid metabolism; (7) butanoate metabolism; (8) citrate cycle; and (9) glycerophospholipid metabolism.

**TABLE 4:** Peak area of metabolites in serum ^1^H-NMR spectra of the HC and MDD groups.

Metabolites	Peak area after normalization	
	**HC**	**MDD**
3-hydroxybutyric acid	0.125 ± 0.99	0.35 ± 1.69
N-acetyl glycoproteins	0.185 ± 0.156	0.41 ± 0.187
Pyruvate	0.125 ± 0.105	0.309 ± 0.147
Choline	0.099 ± 0.011	0.386 ± 0.243
Acetic acid	0.702 ± 0.136	0.455 ± 0.209
Glyceryl	0.756 ± 0.139	0.583 ± 0.205
Acetoacetate	0.082 ± 0.032	0.124 ± 0.389
Lipids	0.343 ± 0.102	0.443 ± 0.114

## 4. Discussion

In clinical practice, BD cases are often misdiagnosed as MDD (22), so finding specific markers to distinguish BD from MDD is crucial. The results of several previous studies have explored the biomarker differences between BD and MDD. For example, one study found that patients with BD and MDD had significantly different features on magnetic resonance imaging (MRI) of the brain, suggesting different neurobiological mechanisms between BD and MDD (23). However, there are still no biomarkers that can accurately distinguish between BD and MDD, and although there are many studies related to the search for biomarkers in the field of metabolomics for BD and MDD, there are only a few reports that include both groups in the study. In this study, we analyzed the serum metabolic phenotypes of BD, MDD, and healthy controls using an NMR metabolomics platform with the aim of exploring whether there are biomarkers that can distinguish BD from MDD. The results showed that the metabolic phenotypes of the BD and MDD groups were significantly different from those of healthy controls, indicating that metabolic changes in those two diseases were significantly different from those in healthy individuals and that there were different metabolite differences between them. Specifically, in the BD group, the levels of 3-hydroxybutyric acid, n-acetyl glycoprotein, β-glucose, pantothenic acid, mannose, glycerol, and lipids were significantly higher than those in healthy controls, while the levels of lactate and acetoacetate were significantly lower than those in healthy controls. In contrast, in the MDD group, the levels of 3-hydroxybutyric acid, n-acetyl glycoprotein, pyruvate, choline, acetoacetic acid, and lipids were significantly higher than those in the healthy control group, while the levels of acetic acid and glycerol were significantly lower than those in the healthy control group.

These differential metabolites above may provide initial insights into the metabolic differences between BD and MDD. Despite the differences in metabolites between MDD and BD, the results of the pathway analysis suggest that most of the most important pathways are shared in these diseases, which is consistent with the findings of previous studies. Notably, most of the key metabolites and their associated pathways appear to focus on three common themes, namely (1) mitochondrial/energy metabolism, (2) neuronal integrity, and (3) signaling/neurotransmission, as described below.

Our study found that lactate and acetoacetate were significantly lower in the BD group. Imbalance in energy homeostasis in BD has been previously reported (24, 25). Previous studies had shown that lactate and acetoacetate were metabolites produced in the process of producing ATP, which plays an important role in many cellular metabolic processes. The results of this study suggest that the energy metabolic status of BD patients may be lower than that of the normal population. Similarly, elevated levels of glycerol and lipids have been associated with the pathogenesis of BD. Previous studies have suggested that dysregulated lipid metabolism in BD patients may be associated with inflammation and brain atrophy. One of these studies found that serum levels of triacylglycerol and cholesterol were significantly higher in BD patients than in controls, whereas HDL levels were significantly lower, which may correlate with the level of inflammation and the degree of brain atrophy in BD patients. Another study also found abnormalities in lipid metabolism in the brain tissue of BD patients, including abnormal accumulation of triglycerides and cholesterol and abnormal degradation and synthesis of myelin proteins in neurons and glial cells. All of these abnormalities may have an impact on neurological function in BD patients, leading to the development of symptoms of mood disorders. Furthermore, Liu et al. (26) found that phospholipid levels were significantly elevated in MDD patients and that these levels were positively correlated with the severity of depression. Consistent with previous studies, we found that abnormal lipid levels were present in both BD and MDD and those glycerolipid metabolic pathways were significantly involved in the development of BD and MDD. Several previous studies have confirmed that disorders or abnormalities of lipid metabolism were associated with neuropsychiatric disorders, such as BD, schizophrenia, and major depressive disorder. Therefore, disorders of lipid metabolism may be an important pathogenesis of BD and MDD and an important cause of the disease.

In addition, the present study found that β-glucose and mannose were significantly elevated in the BD group and that glycolysis/gluconeogenesis was a common metabolic pathway in BD and MDD. Previous studies have shown that there was glucose impairment during some severe psychiatric episodes, even before the start of treatment (27, 28). Recently, extremely high levels of sugar metabolites (sorbitol, gluconate, xylitol, liothymol, arabinitol, and erythritol) and brain glycitol (inositol) have been detected in the brains of patients with bipolar disorder. An autopsy study suggested that abnormal metabolism of sugar and branched-chain amino acids may be a key factor in the pathogenesis of bipolar disorder and that antidiabetic treatment may be beneficial in the treatment of psychiatric disorders (29). Lipid and glucose metabolism was also higher in patients with MDD than in HC (30). Another study found that patients with first-episode depression had significantly higher glucose and triglyceride levels than healthy subjects (31). Singh et al. (32) also suggested that depression was closely associated with diabetes and cerebrovascular disease. Therefore, the use of the glucose-lipid signaling pathway to predict MDD should take diabetes and CVD into account. The absence of abnormal glucose metabolites in the MDD group in our study may be related to its small sample size.

Our study also found that pyruvate and choline were significantly elevated and acetic acid was significantly decreased in the MDD group, while these metabolites were not significantly altered in the BD group. Alterations in choline metabolism are associated with disruptions in intraneural signaling (33, 34), and in addition, the cholinergic hypothesis of depression suggests that cholinergic overactivity and Andrégic hypoactivity lead to depressive states (35). In contrast, choline levels correlate with the clinical state of depression (36, 37), suggesting that choline may be involved in the pathogenesis of depression. As was the case in the present study, a variety of molecules, including acetate and pyruvate, may be MDD-specific drug candidates, and these molecules may lead to more targeted treatment of depressive symptoms, as reported in a review (38). Other studies have also reported that pyruvate levels in the MDD patient group correlate with the severity of depression and may be a potential biomarker candidate for MDD. Pyruvate, as the carboxylate anion of pyruvate, is an end product of glycolysis and can be further involved in the TCA cycle, which is a major process of energy metabolism (39). However, contrary to the results observed in the present study, the review also mentioned that both MDD and BD patients showed a trend of upregulation of choline and lactate in their brains, while the study mentioned that BD serum studies identified seven biomarkers in one or more studies, including pyruvate (38). Lactate and pyruvate were found to be abnormal in BD cerebrospinal fluid biomarkers in more than one study. Therefore, the absence of choline and pyruvate in the BD group in this study may be related to a single metabolic platform, insufficient sample size, and drug effects. In addition, we found that the pyruvate metabolic pathway was significantly, although not significantly, involved in the development of MDD, as well as BD. Therefore, the exact mechanisms of pyruvate pathway alterations in psychiatric disorders need to be further explored. Whether pyruvate, choline, and acetate can serve as potential biomarkers for MDD to distinguish it from BD still needs to be verified by numerous replicated studies in the future. Given that pyruvate provides energy to living cells via the citric acid cycle, our findings also confirm that the aberrant citric acid cycle in mitochondria may be involved in the pathogenesis of BD and MDD.

In the present study, 3-hydroxybutyric acid was the metabolite that jointly distinguishes BD and MDD from HC. In a previous study (40), this biomarker was associated with MDD. 3-hydroxybutyric acid (β-hydroxybutyric acid) is a ketone, a marker known to favor lipid rather than glucose metabolism. 3-hydroxybutyric acid is a ketone elevated in the blood and urine in ketosis. During hypoglycemia, it can be metabolized by the brain for energy (41). Recent studies have shown that 3-hydroxybutyric acid, a metabolite used as a source of energy in the brain, was associated with inflammation of the brain, specifically leading to epilepsy (42). The current data suggest that 3-hydroxybutyrate itself may control the emotional system in the brain through energy metabolite processes (40).

What’s more, we found elevated pantothenic acid levels in the BD group compared to the MDD group, which is consistent with the findings of a previous study that found significantly lower vitamin B12 levels in patients with BD, which may lead to elevated pantothenic acid levels. Pantothenic acid itself is a component of coenzyme a and a precursor of NAA, which is abundant in neurons and is considered an indicator of mitochondrial dysfunction and a marker of neuronal integrity and viability (43, 44). Interestingly, animal studies have shown that levels of NAA are not static and can be reversed by the use of antidepressants, suggesting that antidepressants have neurotrophic effects (45). And whether pantothenic acid can be used as a potential biomarker for BD to distinguish from MDD also needs to be verified in later large-scale studies.

Mays et al. (46) found significant changes in glycine, serine and threonine levels in patients with refractory depression, which is consistent with the findings of the present study that found abnormal glycine, serine and threonine metabolic pathways in MDD subjects, and our previous study also found a high correlation between glycine, serine and threonine metabolism and bipolar depression (47). Glycine or serine combined with glutamate as a coagonist can help activate N-methyl-D-aspartate receptors (NMDARs) (48), and abnormalities in NMDAR activity has been shown to cause affective and cognitive impairment, the core symptom of affective disorders, by decreasing neuroplasticity (49). Reducing serine levels impairs NMDAR-mediated processes in the hippocampus, prefrontal cortex, and amygdala (50, 51), brain structures that are strongly associated with mood disorders (52). We thus hypothesize that this metabolic pathway may be involved in the pathogenesis of mood disorders.

This is only a preliminary study of the differential diagnosis of BD and MDD based on metabolomics biomarkers and serum metabolic pathways, and the results suggest that it is feasible to find biomarkers in the field of metabolomics to differentiate BD and MDD, and also that these differential metabolites may be used as future biomarkers for the diagnosis of BD and MDD in clinical practice. However, the study also has some limitations. First, this study used a small sample size study with a small sample size, which may affect the stability and reliability of the results. Second, other factors, such as diet, body weight, and medication, may also affect the metabolic phenotype of BD and MDD, and the effects of these factors need to be further explored in future studies. Third, it is important to note that the metabolomics platform used in this study may have limitations, as different metabolomics platforms have different sensitivity and accuracy for the detection of different metabolites. Therefore, future studies will need to be validated using multiple metabolomics platforms to further determine the specificity and accuracy of these differential metabolites. Another potential limitation is that our study was a cross-sectional study and could not determine the relationship between metabolite levels and disease progression.

Overall, the present study provides useful clues for finding biomarkers to differentiate BD from MDD and highlights the importance of metabolomics in this field, but more studies are needed to further confirm the reliability of these findings and the potential for clinical application. In addition, a deeper understanding of the pathophysiological mechanisms of BD and MDD is also needed to help us better understand the differences in metabolic phenotypes and thus better identify and treat both diseases.

## 5. Conclusion

In summary, this study used an NMR metabolomics platform to explore the metabolic phenotypic differences between BD and MDD, and found that the metabolic phenotypes of BD and MDD were significantly different from those of healthy controls. Glycerolipid metabolism was significantly involved in BD and MDD. Pyruvate metabolism was significantly involved in MDD. Pyruvate, choline, and acetate may be potential biomarkers for MDD to distinguish from BD, and pantothenic acid may be a potential biomarker for BD to distinguish from MDD. The clinical significance of this study is the discovery of different metabolic phenotypes between BD and MDD, as well as of potential biomarkers for distinguishing BD from MDD. Our findings contribute to the future development of an objective laboratory-based diagnostic test to distinguish between BD and MDD patients, which is very meaningful for the precise diagnosis and treatment of clinical psychiatric disorders. Future studies require more rigorous experimental designs, larger sample sizes, and longitudinal studies to validate our results and conclusions.

## Data availability statement

The datasets presented in this study can be found in online repositories. The names of the repository/repositories and accession number(s) can be found in the article/supplementary material.

## Ethics statement

The studies involving humans were approved by the Medical Ethics Committee of Shanxi Bethune Hospital. The studies were conducted in accordance with the local legislation and institutional requirements. The participants provided their written informed consent to participate in this study. Written informed consent was obtained from the individual(s) for the publication of any potentially identifiable images or data included in this article.

## Author contributions

X-LS, L-NM, and YR contributed to the manuscript preparation. X-LS, L-NM, and Z-ZC performed the data analysis and statistics. JJ and Y-BX oversaw the data/demographic data collection. YW and YR were in charge of design and implementation of the study and contributed to the data interpretation. All authors contributed to the article and approved the submitted version.

## Footnotes

1
http://www.metaboanalyst.ca/

2
http://www.hmdb.ca/

## References

[1] Battle DE. Diagnostic and statistical manual of mental disorders (DSM). *Codas* (2013) 25:191–2. 10.1590/s2317-17822013000200017 24413388

[2] Judd L Akiskal H Schettler P Coryell W Endicott J Maser J A prospective investigation of the natural history of the long-term weekly symptomatic status of bipolar II disorder. *Arch Gen Psychiatry.* (2003) 60:261–9. 10.1001/archpsyc.60.3.261 12622659

[3] Smith DJ Griffiths E Kelly M Hood K Craddock N Simpson SA. Unrecognised bipolar disorder in primary care patients with depression. *Br J Psychiatry.* (2011). 199 49–56. 21292927 10.1192/bjp.bp.110.083840

[4] Angst J. Do many patients with depression suffer from bipolar disorder. *Can J Psychiatry.* (2006). 51:3–5. 16491977 10.1177/070674370605100102

[5] Viktorin A Lichtenstein P Thase ME Larsson H Lundholm C Magnusson PK The Risk of Switch to Mania in Patients with Bipolar Disorder during Treatment with an antidepressant alone and in combination with a mood stabilizer. *Am J Psychiatry.* (2014). 171:1067–73. 24935197 10.1176/appi.ajp.2014.13111501

[6] Williams A Lai Z Knight S Kamali M Assari S McInnis M. Risk factors associated with antidepressant exposure and history of antidepressant-induced mania in bipolar disorder. *J Clin Psychiatry.* (2018) 79:17m11765. 10.4088/JCP.17m11765 29873955

[7] Teneralli R Kern D Cepeda M Gilbert J Drevets W. Exploring real-world evidence to uncover unknown drug benefits and support the discovery of new treatment targets for depressive and bipolar disorders. *J Affect Disord.* (2021) 290:324–33. 10.1016/j.jad.2021.04.096 34020207

[8] Jin H McCrone P. Cost-of-illness studies for bipolar disorder: systematic review of international studies. *Pharmacoeconomics.* (2015) 33:341–53. 10.1007/s40273-014-0250-y 25576148

[9] Patti G Yanes O Siuzdak G. Innovation: metabolomics: the apogee of the omics trilogy. *Nat Rev Mol Cell Biol.* (2012) 13:263–9. 10.1038/nrm3314 22436749 PMC3682684

[10] Xia J Broadhurst D Wilson M Wishart D. Translational biomarker discovery in clinical metabolomics: an introductory tutorial. *Metabolomics.* (2013) 9:280–99. 10.1007/s11306-012-0482-9 23543913 PMC3608878

[11] Beger RD Dunn W Schmidt MA Gross SS Kirwan JA Cascante M Metabolomics enables precision medicine: “A White Paper, Community Perspective”. *Metabolomics.* (2016). 12:149. 10.1007/s11306-016-1094-6 PMC5009152 27642271

[12] Riekeberg E Powers R. New frontiers in metabolomics: from measurement to insight. *F1000Res.* (2017). 6:1148. 10.12688/f1000research.11495.1 PMC5521158 28781759

[13] Dunn W Broadhurst D Atherton H Goodacre R Griffin J. Systems level studies of mammalian metabolomes: the roles of mass spectrometry and nuclear magnetic resonance spectroscopy. *Chem Soc Rev.* (2011) 40:387–426. 10.1039/b906712b 20717559

[14] Ribeiro H Klassen A Pedrini M Carvalho M Rizzo L Noto M A preliminary study of bipolar disorder type I by mass spectrometry-based serum lipidomics. *Psychiatry Res.* (2017) 258:268–73. 10.1016/j.psychres.2017.08.039 28918859

[15] Guo X Jia J Zhang Z Miao Y Wu P Bai Y Metabolomic biomarkers related to non-suicidal self-injury in patients with bipolar disorder. *BMC Psychiatry.* (2022). 22:491. 10.1186/s12888-022-04079-8 35869468 PMC9306041

[16] Guo XJ Wu P Cui XH Jia J Bao S Yu F Pre- and post-treatment levels of plasma metabolites in patients with bipolar depression. *Front Psychiatry.* (2021). 12:747595. 10.3389/fpsyt.2021.747595 34975567 PMC8718604

[17] Guo XJ Xiong YB Jia J Cui XH Wu WZ Tian JS Altered metabolomics in bipolar depression with gastrointestinal symptoms. *Front Psychiatry.* (2022). 13:861285. 10.3389/fpsyt.2022.861285 35686183 PMC9170992

[18] Takeshima M Oka T. DSM-5-defined “mixed features” and Benazzi’s mixed depression: which is practically useful to discriminate bipolar disorder from unipolar depression in patients with depression? *Psychiatry Clin Neurosci.* (2015). 69:109–16. 24902989 10.1111/pcn.12213

[19] Zhu Y Wu X Liu H Niu Z Zhao J Wang F Employing biochemical biomarkers for building decision tree models to predict bipolar disorder from major depressive disorder. *J Affect Disord.* (2022) 308:190–8. 10.1016/j.jad.2022.03.080 35439462

[20] Huang KL Chen MH Hsu JW Tsai SJ Bai YM. Using classification and regression tree modeling to investigate appetite hormones and proinflammatory cytokines as biomarkers to differentiate bipolar I depression from major depressive disorder. *CNS Spectr.* (2021). [Epub ahead of print]. 10.1017/S109285292100016X 33563365

[21] Ren J Zhao G Sun X Liu H Jiang P Chen J. Identification of plasma biomarkers for distinguishing bipolar depression from major depressive disorder by iTRAQ-coupled LC-MS/MS and bioinformatics analysis. *Psychoneuroendocrinology.* (2017). 86:17–24. 28910601 10.1016/j.psyneuen.2017.09.005

[22] Mota R Gazal M Acosta B de Leon P Jansen K Pinheiro R Interleukin-1β is associated with depressive episode in major depression but not in bipolar disorder. *J Psychiatr Res.* (2013) 47:2011–4. 10.1016/j.jpsychires.2013.08.020 24074516

[23] Kempton M Salvador Z Munafò M Geddes J Simmons A Frangou S Structural neuroimaging studies in major depressive disorder. Meta-analysis and comparison with bipolar disorder. *Arch Gen Psychiatry.* (2011) 68:675–90. 10.1001/archgenpsychiatry.2011.60 21727252

[24] Yoshimi N Futamura T Kakumoto K Salehi AM Sellgren CM Holmén-Larsson J Blood metabolomics analysis identifies abnormalities in the citric acid cycle, urea cycle, and amino acid metabolism in bipolar disorder. *BBA Clin.* (2016) 5:151–8. 27114925 10.1016/j.bbacli.2016.03.008 PMC4832124

[25] Mansur RB Lee Y McIntyre RS Brietzke E. What is bipolar disorder? A disease model of dysregulated energy expenditure. *Neurosci Biobehav Rev.* (2020). 113:529–545. 32305381 10.1016/j.neubiorev.2020.04.006

[26] Liu X Li J Zheng P Zhao X Zhou C Hu C Plasma lipidomics reveals potential lipid markers of major depressive disorder. *Anal Bioanal Chem.* (2016) 408:6497–507. 10.1007/s00216-016-9768-5 27457104

[27] Calkin CV Gardner DM Ransom T Alda M. The relationship between bipolar disorder and type 2 diabetes: more than just co-morbid disorders. *Ann Med.* (2013) 45:171–81. 22621171 10.3109/07853890.2012.687835

[28] Garcia-Rizo C Kirkpatrick B Fernandez-Egea E Oliveira C Bernardo M. Abnormal glycemic homeostasis at the onset of serious mental illnesses: a common pathway. *Psychoneuroendocrinology.* (2016). 67:70–5. 26878465 10.1016/j.psyneuen.2016.02.001 PMC4844848

[29] Zhang R Zhang T Ali A Al Washih M Pickard B Watson D. Metabolomic profiling of post-mortem brain reveals changes in amino acid and glucose metabolism in mental illness compared with controls. *Comput Struct Biotechnol J.* (2016) 14:106–16. 10.1016/j.csbj.2016.02.003 27076878 PMC4813093

[30] Zheng H Zheng P Zhao L Jia J Tang S Xu P Predictive diagnosis of major depression using NMR-based metabolomics and least-squares support vector machine. *Clin Chim Acta.* (2017) 464:223–7. 10.1016/j.cca.2016.11.039 27931880

[31] Xia Q-C Wang G-H Wang H-L Xie Z-B Fang Y Li Y. Study of metabolism of glucose and lipid in patients with first-episode depression. *J Clin Psychiatry.* (2009) 19:241–3.

[32] Singh P Khullar S Singh M Kaur G Mastana S. Diabetes to cardiovascular disease: is depression the potential missing link? *Med Hypotheses.* (2015) 84:370–8. 10.1016/j.mehy.2015.01.033 25655224

[33] Exton J. Signaling through phosphatidylcholine breakdown. *J Biol Chem.* (1990) 265:1–4. 2104616

[34] Moore CM Breeze JL Gruber SA Babb SM Frederick BB Villafuerte RA Choline, myo-inositol and mood in bipolar disorder: a proton magnetic resonance spectroscopic imaging study of the anterior cingulate cortex. *Bipolar Disord.* (2000) 2(3 Pt. 2) 207–16. 11249799 10.1034/j.1399-5618.2000.20302.x

[35] Janowsky D el-Yousef M Davis J Sekerke H. A cholinergic-adrenergic hypothesis of mania and depression. *Lancet.* (1972) 2:632–5. 10.1016/s0140-6736(72)93021-8 4116781

[36] Breisch J Averhoff B. Identification of osmo-dependent and osmo-independent betaine-choline-carnitine transporters in Acinetobacter baumannii: role in osmostress protection and metabolic adaptation. *Environ Microbiol.* (2020) 22 2724–2735. 32219961 10.1111/1462-2920.14998

[37] Lin JC Lee MY Chan MH Chen YC Chen HH. Betaine enhances antidepressant-like, but blocks psychotomimetic effects of ketamine in mice. *Psychopharmacology.* (2016) 233 3223–35. 27363702 10.1007/s00213-016-4359-x

[38] MacDonald K Krishnan A Cervenka E Hu G Guadagno E Trakadis Y. Biomarkers for major depressive and bipolar disorders using metabolomics: a systematic review. *Am J Med Genet.* (2019) 180:122–37. 10.1002/ajmg.b.32680 30411484

[39] Kładna A Marchlewicz M Piechowska T Kruk I Aboul-Enein H. Reactivity of pyruvic acid and its derivatives towards reactive oxygen species. *Luminescence.* (2015) 30:1153–8. 10.1002/bio.2879 25754627

[40] Setoyama D Kato T Hashimoto R Kunugi H Hattori K Hayakawa K Plasma metabolites predict severity of depression and suicidal ideation in psychiatric patients-A multicenter pilot analysis. *PLoS One.* (2016) 11:e0165267. 10.1371/journal.pone.0165267 27984586 PMC5161310

[41] Akram M. A focused review of the role of ketone bodies in health and disease. *J Med Food.* (2013) 16:965–7. 10.1089/jmf.2012.2592 24138078

[42] Sada N Lee S Katsu T Otsuki T Inoue T. Epilepsy treatment. Targeting LDH enzymes with a stiripentol analog to treat epilepsy. *Science.* (2015) 347:1362–7. 10.1126/science.aaa1299 25792327

[43] Chen L Dai H Dai Z Xu C Wu R. Anterior cingulate cortex and cerebellar hemisphere neurometabolite changes in depression treatment: a ^1^H magnetic resonance spectroscopy study. *Psychiatry Clin Neurosci.* (2014) 68:357–64. 10.1111/pcn.12138 24393367

[44] Tallan H Moore S Stein W. N-Acetyl-L-aspartic acid in brain. *J Biol Chem.* (1956) 219:257–64. 13295277

[45] Moffett JR Ross B Arun P Madhavarao CN Namboodiri AM. N-Acetylaspartate in the CNS: from neurodiagnostics to neurobiology. *Prog Neurobiol.* (2007). 81:89–131. 17275978 10.1016/j.pneurobio.2006.12.003 PMC1919520

[46] Maes M Verkerk R Vandoolaeghe E Lin A Scharpé S. Serum levels of excitatory amino acids, serine, glycine, histidine, threonine, taurine, alanine and arginine in treatment-resistant depression: modulation by treatment with antidepressants and prediction of clinical responsivity. *Acta Psychiatr Scand.* (1998) 97:302–8. 10.1111/j.1600-0447.1998.tb10004.x 9570492

[47] Ren Y Bao S Jia Y Sun X Cao X Bai X Metabolic profiling in bipolar disorder patients during depressive episodes. *Front Psychiatry.* (2020) 11:569612. 10.3389/fpsyt.2020.569612 33391044 PMC7772141

[48] Meunier C Dallérac G Le Roux N Sacchi S Levasseur G Amar M Serine and glycine differentially control neurotransmission during visual cortex critical period. *PLoS One.* (2016) 11:e0151233. 10.1371/journal.pone.0151233 27003418 PMC4803205

[49] Peyrovian B Rosenblat J Pan Z Iacobucci M Brietzke E McIntyre R. The glycine site of NMDA receptors: a target for cognitive enhancement in psychiatric disorders. *Prog Neuropsychopharmacol Biol Psychiatry.* (2019) 92:387–404. 30738126 10.1016/j.pnpbp.2019.02.001

[50] Fossat P Turpin FR Sacchi S Dulong J Shi T Rivet JM Glial D-serine gates NMDA receptors at excitatory synapses in prefrontal cortex. *Cereb Cortex.* (2012). 22 595–606. 21690263 10.1093/cercor/bhr130

[51] Curcio L Podda MV Leone L Piacentini R Mastrodonato A Cappelletti P Reduced D-serine levels in the nucleus accumbens of cocaine-treated rats hinder. *Brain.* (2013). 136(Pt. 4):1216–30. 23518710 10.1093/brain/awt036

[52] Castanheira L Silva C Cheniaux E Telles-Correia D. Neuroimaging correlates of depression-implications to clinical practice. *Front Psychiatry.* (2019) 10:703. 10.3389/fpsyt.2019.00703 31632306 PMC6779851

